# Intracarotid Transplantation of Skin-Derived Precursor Schwann Cells Promotes Functional Recovery After Acute Ischemic Stroke in Rats

**DOI:** 10.3389/fneur.2021.613547

**Published:** 2021-02-04

**Authors:** Jingjing Liang, Ronghui Cui, Jinglei Wang, Jiabing Shen, Ying Chen, Maosheng Cao, Kaifu Ke

**Affiliations:** ^1^Department of Neurology, Affiliated Hospital of Nantong University, Nantong, China; ^2^Research Center of Clinical Medicine, Affiliated Hospital of Nantong University, Nantong, China; ^3^Nantong University, Nantong, China

**Keywords:** skin-derived precursors, conditioned medium, neuron, ischemic stroke, neuroprotection

## Abstract

**Purpose:** Skin-derived Precursor Schwann cells (SKP-SCs) have been reported to provide neuroprotection for the injured and dysmyelinated nervous system. However, little is known about SKP-SCs on acute ischemic stroke (AIS). We aimed to explore the efficacy and the potential mechanism of action of SKP-SCs on AIS in a rat ischemic stroke model.

**Methods:** Adult male Sprague–Dawley rats were subjected to a middle cerebral artery occlusion (MCAO) for 1.5 h on Day 0 and subsequently received an intracarotid injection of 2 × 10^6^ green fluorescent protein (GFP) -labeled SKP-SCs or phosphate buffered saline (PBS) during reperfusion. Neurological function was assessed by behavioral tests on Days 1, 4, 7, 14, and 28. In a satellite cohort, rat brains were harvested and infarct volume was measured with 2,3,5-triphenyltetrazolium chloride (TTC) staining on Days 1 and 7, and migration and survival of SKP-SCs in the brain were traced by monitoring green fluorescence at 6 and12 h on Day 0, and on Days 1, 4, 7, 14, and 28. Histopathology and immunofluorescence staining were used to analyze the morphology, survival and apoptosis of neurons. Additionally, in an *in vitro* SKP-SC co-culture model using fetal rat primary cortical neurons underwent oxygen glucose deprivation/reoxygenation (OGD/R), Western blot was used to detect the expression of apoptosis indicators including activated caspase-3, Bax, and Bcl-2. TUNEL staining was used to count apoptotic cells.

**Results:** Intracarotid transplantation of SKP-SCs effectively migrated to the periinfarct area and survived for at least 4 weeks. Transplanted SKP-SCs inhibited neuronal apoptosis, reduced infarct volume, and improved neurological recovery in the MCAO rats. Moreover, *in vitro* data showed that SKP-SCs treatment inhibited OGD/R-induced neuronal apoptosis and promoted survival of the cultured primary cortical neurons.

**Conclusions:** Intracarotid transplantation of SKP-SCs promoted functional recovery in the rat AIS model and possesses the potential to be further developed as a novel therapy to treat ischemic stroke in humans.

## Background

Stroke is the leading cause of neurological disability in adults worldwide (1), with the majority of patients (~87%) being ischemic in etiology due to the occlusion of cerebral artery or arteries (2). Although intravenous thrombolysis and endovascular treatment have been proven to be beneficial, hospital-sourced databases demonstrate that the thrombolytic rate of patients with acute ischemic stroke (AIS) is only between 3.4 and 9.1%, and treatment rate of endovascular therapy is even lower (2). Many patients are just not eligible for these treatments due to key exclusion criteria (e.g., time after onset and partial vascular occlusion). Currently, only about 13%-20% AIS patients meet the indications for endovascular mechanical thrombectomy. Among these patients, only <50% of the patients can achieve a good clinical outcome (3). Therefore, there is an unmet need for new interventions to bring or enhance the efficacy in an extended treatment time window.

Studies have shown that recovery/compensation of neural functions after stroke could actually last for months, depending on the plastic environment of the ischemic brain and the degree of neural circuit remodeling (4). This provides a solid scientific foundation for interventions in an extended time window beyond the time allowed for thrombolysis or thrombectomy. A growing body of evidence suggested that stem cells could offer potential benefits as a novel therapeutic method during this extended time window. Stem cells have been shown to improve functional prognosis after stroke possibly through brain plasticity mechanisms such as axon germination, synaptic remodeling, remyelination, angiogenesis, or neurogenesis, depending on cell types used. For example, bone marrow-derived endothelial progenitor cells and circulating CD34+ hematopoietic stem cells have strong angiogenic capacity (5). Mesenchymal stem cells, especially adipose-derived mesenchymal stem cells, have anti-inflammatory and immunoregulatory capabilities (5). Neural stem cells are immature self-renewing cells that can differentiate into neurons and glial cells in the periinfarct area.

Skin-derived precursors (SKPs) are dermally derived pluripotent precursor cells that are isolated and cultured from murine dorsal skin or human foreskin (6–8). These mesodermally derived SKPs can respond to neural crest cues such as forskolin, heregulin-1β or N2 supplement to generate functional Schwann cells without genetic manipulation (9). Previous studies demonstrated that transplantation of peripheral nerve Schwann cells can provide both trophic support for spared axons and participate in remyelination of the injured spinal cord (9–13). However, one of the shortcomings of this therapy is that it requires surgical biopsies to harvest adult nerves. Therefore, skin-derived precursor Schwann cells (SKP-SCs) could help avoid such an invasive procedure. SKP-SCs are highly similar at the transcriptional level to the peripheral nerve Schwann cells (14). The transplanted SKP-SCs in the normal or injured central nervous system environment can survive and maintain its peripheral phenotype for at least 5 weeks (7). In the previous reports, we found that SKP-SCs protected SH-SY5Y cells against 6-OHDA-induced neurotoxicity (15), and bridged rat sciatic nerve gap by generating an acellular matrix to modify chitosan/silk scaffolds (16).

We hereby hypothesize that SKP-SCs will have therapeutic effect on ischemic stroke by improving the disruption of neural circuitry and suppressing ischemia-induced apoptosis in the infarcted brain region. To test this hypothesis, we have transplanted SKP-SCs via a novel intracarotid route during reperfusion in a transient rat middle cerebral artery occlusion (MCAO) model. We found that SKP-SCs inhibited apoptosis, reduced infarct size, and improved neural functional recovery in behavioral test.

## Methods

### Experimental Animals

All protocols of in the present animal studies were reviewed and approved by the Institutional Animal Use and Care Committee of Nantong University, in accordance with the Guide for the Care and Use of Laboratory Animals (NIH publication No. 85-23, National Academy Press, Washington, DC, USA, revised 1996). Standard biosafety and the institutional standard operating procedures were strictly followed in SKP-SCs and all cell culture experiments. Healthy male Sprague-Dawley (SD) rats were housed in cages on a 12-h light/dark cycle. Food and water were available *ad libitum*. After at least 3 days habituation, rats were randomly assigned into three groups: sham operation, MCAO + PBS and MCAO + SKP-SCs. The sample size estimation for behavioral studies was based on the effects of SKP-SCs in our previous experiment (15). A sample size of 10 rats per group was deemed adequate.

### MCAO Procedures

Transient MCAO was induced by intraluminal vascular occlusion surgery following the method previously described (17). Briefly, rats were anesthetized with a mixed solution containing ketamine (100 mg/kg; sigma) and xylazine (10 mg/kg; sigma) (i.p.). The right common carotid artery (CCA), external carotid artery (ECA), and internal carotid artery (ICA) were dissected through the midline cervical incision. A poly-lysine coated monofilament nylon suture (Beijing Cinontech Co., China) was introduced from the ECA into the ICA and inserted up until the middle cerebral artery was occluded. After 1.5 h of occlusion, the suture was withdrawn to achieve reperfusion. During the surgery, the temperature of the rats was maintained at 37.0 ± 0.5°C using a heating pad. Rats in the sham group underwent the same surgical procedures as above, except that suture for artery occlusion was not inserted into the ICA. A five-point scale was used to evaluate the neurological deficits when the animals attained full consciousness following MCAO surgery on Day 0: 0. no observable deficits; (1) unable to extend contralateral forepaw; (2) circling toward contralateral to infarct; (3) falling toward contralateral to infarct; (4) low level of consciousness with no spontaneous movement. Only MCAO animals with the scores of 1–3 were included for further behavioral and histological evaluations. Thirteen percent of the rats underwent MCAO surgery were excluded for not meeting the inclusion criterion on Day 0. Death was observed on Day 1 and afterwards in the MCAO animals. The total mortality rate was 39.4% that was approximately equally distributed in the PBS control group (38.5%) and the SKP-SCs group (41.2%). All animals with sham surgery after attaining full consciousness survived and showed no neurological deficits.

### SKP-SCs Isolation, Expansion, and Transplantation

Isolation and expansion of SKP-SCs were performed following the protocol reported by Biernaskie et al. (8). Acquired SKP-SCs were cultured for 5 days in DMEM/F12 (3:1) medium supplemented with 1% FBS, 2% N2 supplement, 5 uM Forskolin, 50 ng/ml Heregulin-1β, referred as Schwann cell differentiation medium. Intracarotid transplantation of SKP-SCs was initiated at 1.5 h after MCAO. Right before the transplantation, a microvascular clip was used to temporarily block the CCA blood flow. A polyethylene tube (PE-10, Shenzhen Ruiward Life Technology Co., China) was inserted from the ECA into the ICA, and 50 μl of cell suspension (containing 2 × 10^6^ cells) was carefully infused through the tube for 10 min in MCAO + SKP-SCs group. This dose was selected based on the results of previous studies (15, 16). Reperfusion was resumed after the completion of SKP-SCs infusion. Rats in the MCAO + PBS group underwent the same surgical procedures, but received only 50 μl of PBS. Vital signs of all rats were monitored and found to be stable and no severe bleeding had occurred during the procedure. None of the rats received immunosuppressants.

### Behavioral Tests

Behavioral tests were performed on all rats on Days 1, 4, 7, 14, and 28 after MCAO (Day 0). Two investigators designated for the test were blinded to both the surgery and transplantation. Sensorimotor deficits were evaluated by improved neurological deficit score (mNSS) and rotarod. Weight changes in rats were also recorded.

mNSS is an 18-point scoring system that includes movement (muscular state and abnormal movement), sensation (visual, tactile and proprioceptive), reflex (auricle, cornea, shock) and balance assessments. Higher scores indicate more severe neurological damage.

Motor coordination and balance were assessed by rotarod test. The fatigue rotator (Shenzhen Ruiward Life Technology Co., China) accelerates from 4 to 40 rpm in 5 min. The length of time the rats remained on the smooth rotating rod was measured. Rats were trained for a consecutive 3 days before MCAO procedures to adapt to the rod-rotator. The maximal exercise time for each training/test was 300 s. Only rats that were able to stay on rotarod for 290–300 s were included in the experiment. The results acquired 1 day before surgery was recorded as baseline. At the time point of postoperative behavioral test, three measurements with an interval of more than 5 min were recorded, and the average was calculated.

Baseline body weight of rats was measured and recorded 1 day before surgery, and rats with the weight of 190–220 g were included in the experiment. Body weight of each rat was recorded before behavioral tests were performed at each time point.

### Infarct Volume Assessment

Rats from a satellite cohort were sacrificed 1 day and 7 days after SKP-SCs transplantation, and cerebral infarction volume was measured after 2,3,5-triphenyltetrazolium chloride (TTC) staining. Briefly, after deep anesthesia, rats were transcardially perfused with saline. The brains were quickly removed, and the coronal slices cut at 2 mm with a rat brain mold (Shenzhen Ruiward Life Technology Co., China). These slices were immediately stained with 2% TTC solution (Sigma, USA) in PBS for 30 min in a 37°C incubator. Normal brain tissue is stained red, while infarcted areas remain unstained. The size of the infarcted area was measured by Image J software (NIH, USA) and the infarct volume is expressed as the percentage of the infarcted brain volume in the lesioned (right, ipsilateral) hemisphere over the intact left, contralateral hemisphere (*total brain volume of the intact left (contralateral) hemisphere minus the leftover healthy brain volume of the right (ipsilateral) hemisphere, divided by the total volume of the left hemisphere*).

### Histopathology, Immunofluorescence, and TUNEL Assay

Rats from the satellite cohort at 6 h, 12 h on Day 0, Days 1, 4, 7, 14, and 28 were deeply anesthetized with ketamine (100 mg/kg; sigma) and xylazine (10 mg/kg; sigma) (i.p.) and perfused transcardially with 4% paraformaldehyde in 0.1 M phosphate buffer following a quick 0.9% NaCl saline flush. After perfusion, the brains were quickly removed and post-fixed for 24 h in the same fixative, followed by immersion in 20 and 30% sucrose. Coronal brain sections were cut at 12 μm with a Leica cryostat and mounted on slides precoated with adhesive. After drying at room temperature, the slides were boxed and stored at−20°C before use.

#### Hematoxylin and Eosin (H&E) Staining

Histopathologic changes of the cortex and striatum in the penumbra area were studied using H&E (Beyotime, China) staining in rats of Day 4. After fixation, the sections were stained in hematoxylin solution for 30 s and eosin solution for 1 min. Following gradient dehydration, the slides were further cleared with xylene and coverslipped with neutral gum. The number and morphology of neurons were examined under microscope.

#### Nissl Staining

Nissl staining was used in rats of Day 4 to further observe pathologic changes of neurons in the penumbra. After being thawed to room temperature, slides were rinsed with distilled water for 5 min to remove the gel around the tissue. Staining was achieved by immersing the slides in the Nissl Staining Solution (Beyotime, China) for 10–20 min at room temperature. After brief rinsing, all slides went through ascending dehydration and were cleared in xylene before being coverslipped.

#### Fluorescence Tracing and Immunofluorescence

Tissues of MCAO+ SKP-SCs group were directly observed under a fluorescence microscope (ZEISS, Germany) to detect survival and migration of GFP-labeled SKP-SCs in the rat brain at 6 h, 12 h on Day 0, Days 1, 4, 7, 14 and 28 after MCAO. To further observe the health of neurons and glial cells, as well as neuronal dendritic processes and axonal growth, additional sets of slides were stained with antibodies against different neuronal and glial cell markers, including NeuN, GFAP, MAP2, and GAP43. After temperature equilibration and drying, sections mounted on slides were washed with PBS for 10 min, then blocked with 0.1% Triton x-100 for 2 h, before being incubated overnight at 4°C with the following antibodies: rabbit anti-NeuN antibody (1:500, Abcam, USA) and rabbit anti-GFAP antibody (1:500, Abcam, USA), mouse anti-MAP2 antibody (1:500, Abcam, USA), and rabbit anti-GAP43 antibody. Indirect fluorescence by incubating sections at room temperature for 2 h with goat-anti-rabbit IgG conjugated with Alexa Fluor 594 or goat-anti-mouse-IgG conjugated with Alexa Fluor 488 (1:1000, Life Technologies) was used for detection. Sections were further incubated with Hoechst for 10 min before being coverslipped for microscopic observation.

#### TUNEL Assay

TUNEL assay was used to examine the apoptosis in the penumbra. After taken out of box, slides were dried at 37°C for 1 to 2 h and rinsed with PBS before being post-fixed with 4% paraformaldehyde for 30 min. Following block with 1% Triton X-100 for 10 min, 50 μl of TUNEL test solution (Sigma, USA) was applied to each slide and incubated for 1 h at room temperature. After that, slides were further counterstained with Hoechst for 5 min. Apoptosis index was calculated by counting the total number of TUNEL positive cells and all the number of Hoechst positive cells.

**Figure 1:**
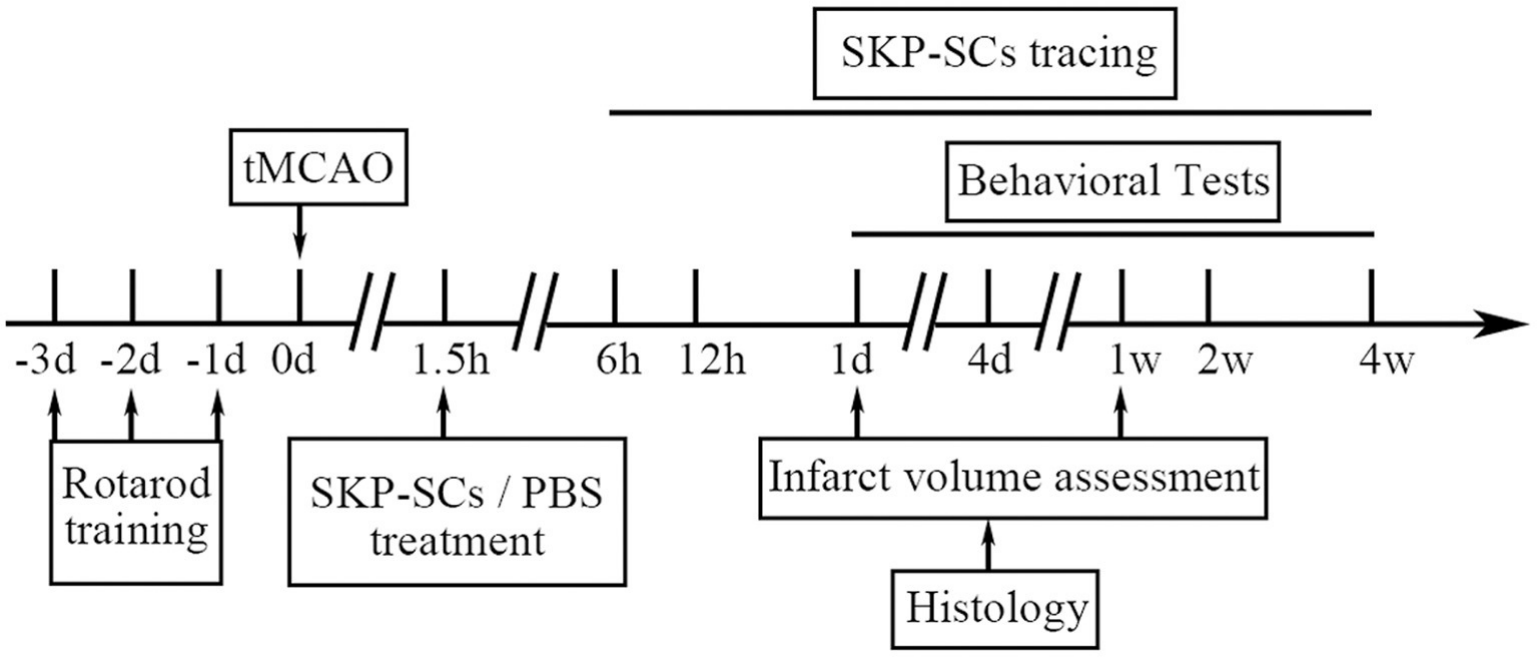
Study design of the *in vivo* study. d, day; h, hour; w, week.

The whole *in vivo* study design is illustrated in Figure 1.

### *In vitro* Assay

To explore the mechanism of action that SKP-SCs salvage injured neurons in the penumbra region after ischemia, an *in vitro* co-culture experiment using rat primary cortical neurons (PCNs) and SKP-SCs was designed and performed.

#### Extraction and Culture of PCNs

Timed pregnant SD rats were anesthetized with ether and fetuses (E17) were removed from the uterus. Fetal brains were quickly removed and placed in pre-chilled DMEM medium. Cerebral cortex was isolated and cut it into pieces. After digestion with 0.25% trypsin at 37°C for 7 min, DMEM containing 10% fetal bovine serum was added. Homogenization was achieved by repeated pipetting. The suspension was then centrifuged at 12,000 rpm for 5 min and cells resuspended in DMEM with 10% fetal bovine serum and filtered. Cells were then seeded on cell culture plates and petri dishes, with the inoculation density of 5 × 10^4^ in a 24-well plate, 1 × 10^5^ in a 96-well plate, and 3 × 10^6^ in a medium dish. Once cells adhered to the wall after culture for 4 to 6 h, the culture medium was replaced with the whole culture medium (Neurobasal + 2% B27 + 1% L glutamine + 1% antibiotics).

#### Oxygen Glucose Deprivation/Reoxygenation (OGD/R)

OGD/R procedures were used to simulate the effect of MCAO and reperfusion on neurons *in vivo*. After replacing Neurobasal medium containing B27, L-glutamine, and antibiotics with glucose-free DMEM medium, the PCNs culture plate was placed in a 37°C hypoxia incubator for 1.5 h. Once the oxygen-glucose deprivation was completed, the cell culture solution was replaced with Neurobasal medium and reoxygenated, and further cultured in a regular cell culture incubator at 37 °C with 20% oxygen and 5% CO2.

#### Preparation of SKP-SC Conditioned Medium (CM)

DMEM/F12 FBS-free medium was used to prepare SKP-SC CM when SKP-SCs density reached ~80%. After further culture for 48 h, SKP-SCs CM were collected and concentrated using a centrifugal filtration device (3K Amicon Ultra, Millipore) at 3000 × g. SKP-SCs-free control medium was prepared in the same way. Concentrated (about 50-fold) CM samples were quickly frozen and stored at −80°C until use.

#### Cell Viability

Cell viability was assessed with a Cell Counting Kit-8 assay kit (CCK-8) (Dojindo, Japan). PCNs were cultured for 6 days and treated with or without OGD for 1.5 h followed by addition of SKP-SCs CM or control medium for co-culture for another 24 h during reoxygenation. After adding 10 μl of CCK-8 solution to each well, the 96-well plates were incubated in a cell incubator for 1.5 h and the absorbance was measured at 450 nm with a microplate reader (BioTek, USA).

#### Western Blot

Before adding CCK-8 assay kit, some of the above co-cultured PCNs were harvested. These cells were lysed with Cell Lysis (Invitrogen, USA) to obtain whole-cell lysate. BCA protein assay kit (Beyotime, China) was used to measure protein concentration. Twenty micrograms of protein at the concentration of 4 μg/μl was loaded to each well of SDS-PAGE gel (Beyotime, China) for electrophoresis, and the separated proteins were transferred to PVDF (Millipore, USA) membranes. After blocking with 5% skim milk at room temperature for 2 h, the membranes were incubated with the following primary antibody at 4°C overnight: cleaved caspase-3 (1: 200, Cell Signaling Technology, USA), Bax (1: 200, Abcam, UK), Bcl-2 (1: 200 (Abcam, UK), and GAPDH (1: 200, Abcam, UK). The membranes were further incubated with an HRP-conjugated secondary antibody at room temperature for 2 h and reacted with enhanced chemiluminescence (ECL) agent (Pierce, USA). Protein signals were detected by an automatic chemiluminescence/fluorescence image analysis system (Tanon, China). Quantification was performed using Image J software (NIH, USA).

### Statistical Analysis

GraphPad Prism 5.0 (GraphPad, La Jolla, CA, USA) software was used for statistical data analysis. All data were presented as mean ± SEM. Student's *t* test was used for pairwise comparisons. A two-sided one-way ANOVA and *post hoc* Bonferroni test were used to compare multiple groups. A value of *p* < 0.05 was considered statistically significant.

## Results

### Characterization of Schwann Cell Phenotype

**Figure 2:**
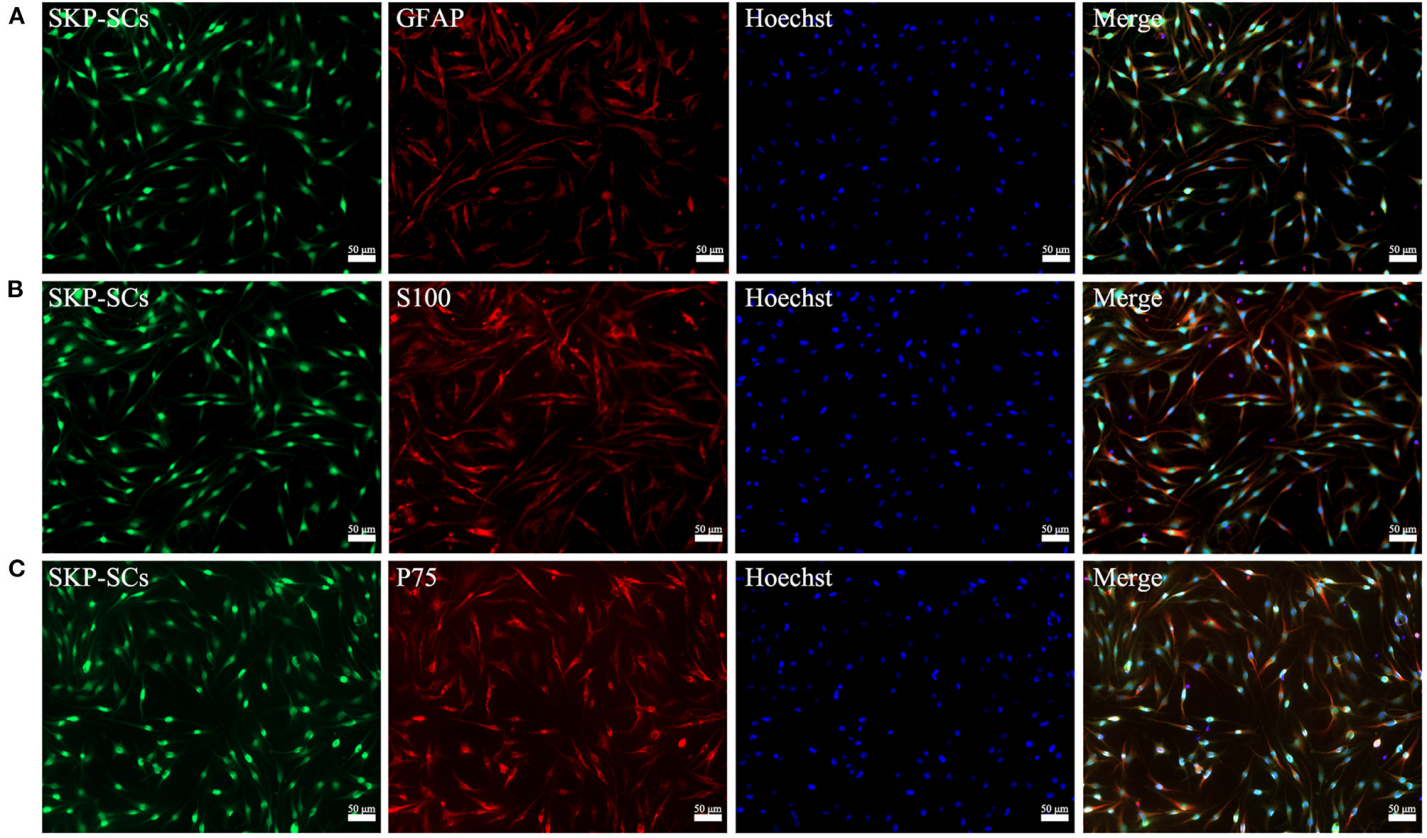
Characterization of GFP-labeled SKP-SCs (green, **A–C**). Immunofluorescence staining showed that SKP-SCs were positive for GFAP (red, **A**), S100 (red, **B**), and P75 (red, **C**). All cells were counterstained with Hoechst nuclear fluorescent dye (blue, **A–C**). All images were merged to demonstrate colocalization of green fluorescence, red immunoreactivity and blue nuclei. Scale bars = 50 μm.

Schwann cell phenotype of the SKP-SCs was characterized before transplanting. SKP-SCs showed typical bipolar morphology of Schwann cells (Figures 2A–C). Immunofluorescence staining confirmed that SKP-SCs were positive for Schwann cell lineage markers S100, GFAP and P75 (Figures 2A–C).

### Migration and Survival of SKP-SCs After Intracarotid Transplantation

**Figure 3:**
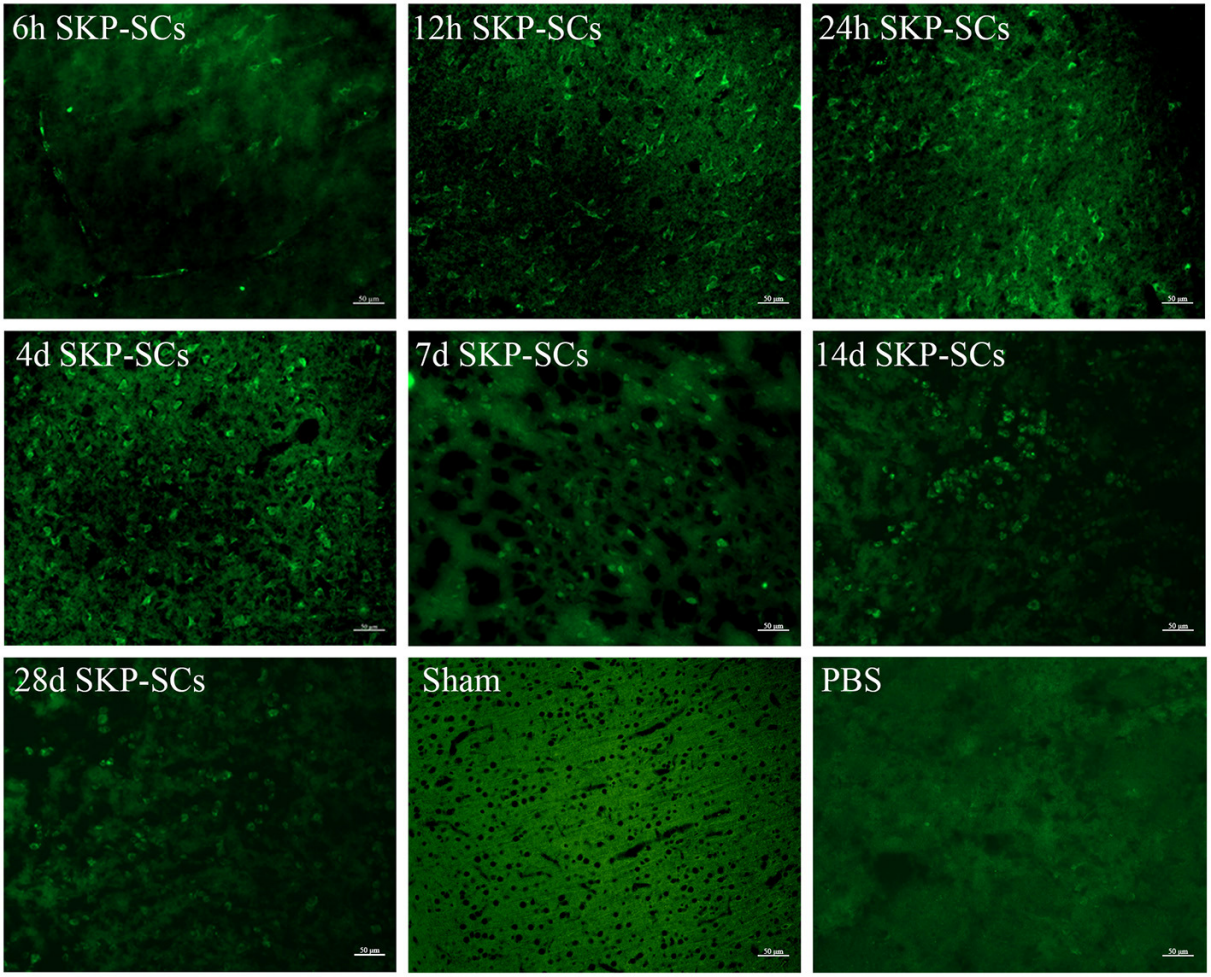
Survival and migration of SKP-SCs after intracarotid transplantation. Starting from 6 h after middle cerebral artery occlusion, SKP-SCs (green) were seen in the penumbra but a good portion of SKP-SCs were still in the vasculature. At 12 h, all SKP-SCs appeared to migrate into the penumbra and survived there for at least 28 days. Scale bars = 50 μm.

SKP-SCs were clearly detected in the ischemic region of the right cerebral hemisphere as early as 6 h after transplantation, although some SKP-SCs remained in the blood vessels (Figure 3, 6 h). Throughout all time points observed, SKP-SCs were mainly concentrated in the ipsilateral infarct area, no SKP-SCs were detected in the ipsilateral healthy brain tissue or the healthy, contralateral hemisphere. SKP-SCs were continuously detectable during the 28 days observation and appeared to survive for at least 28 days in the ipsilateral cerebral penumbra after transplantation (Figure 3, 12 h, 24 h, 4 d, 7 d, 14 d, 28 d).

### Effects of SKP-SCs Transplantation on the Infarct Volume and Neurological Function

**Figure 4:**
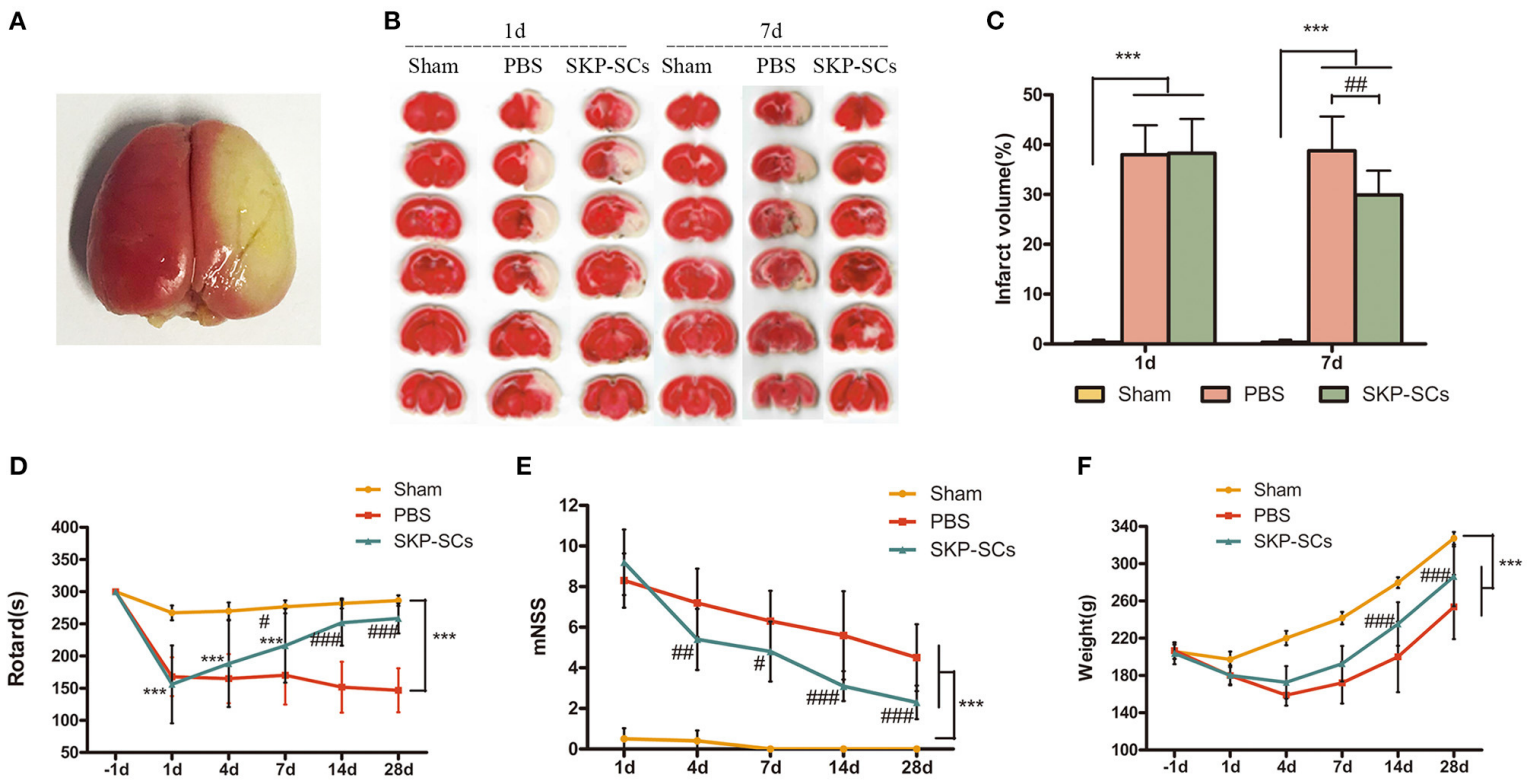
Efficacy of SKP-SCs transplantation on infarct volume and neurological functions in the rat MCAO model. Whole brain TTC staining 1 day after the surgery **(A)** and brain slices stained with TTC from rats of groups of Sham, MCAO+PBS, and MCAO+SKP-SCs **(B)**. Quantitative analysis showed a significant reduction in infarct volume by SKP-SCs on Day 7 **(C)**. Starting from Day 7, significantly improved Rotarod performance was observed by SKP-SCs. The performance continued to be improved throughout the test period until Day 28 **(D)**. Similarly, modified neurological deficit score (mNSS) assessment showed a continuous improvement starting from Day 4 after cerebral artery occlusion **(E)**. Comparing with the PBS controls, SKP-SCs improved body weight as observed on Day 14 and Day 28 **(F)**. *N* = 10 in each group, ***p < 0.001 compared with Sham group. ^#^*p* < 0.05, ^*##*^*p* < 0.01, ^*###*^*p* < 0.001 compared with MCAO + PBS group.

TTC staining demonstrated a remarkable infarction in the right hemisphere (Figures 4A,B). SKP-SCs transplantation appeared to have no effect on Day 1 but significantly reduce infarct volume on Day 7. Infarct volume was reduced from 38.78 to 29.91% (*p* < 0.01), accounting for a 22.87% reduction (Figure 4C).

Neurological recovery was measured using rotarod and mNSS tests. Starting from Day 7, SKP-SCs showed a significant improvement in rotarod time and continued to improve toward a full functional recovery on Day 14 and Day 28 (*p* < 0.001). In contrast, PBS control displayed no effect (Figure 4D). Consistent with the rotarod results, SKP-SCs significantly lowered mNSS scores from Day 4 throughout Day 28 (*p* < 0.05 ~ 0.001) (Figure 4E). The mean values of body weight of the rats in the SKP-SCs group and the PBS control group were comparable in the initial phase, but starting on Day 14, rats receiving SKP-SCs gained more body weight (*p* < 0.001) (Figure 4F). Collectively, SKP-SCs transplantation significantly reduced the infarct volume and improved the neurological recovery in the current rat MCAO stroke model.

### Neuroprotective Effect of SKP-SCs Transplantation on Neurons Around the Infarct

**Figure 5:**
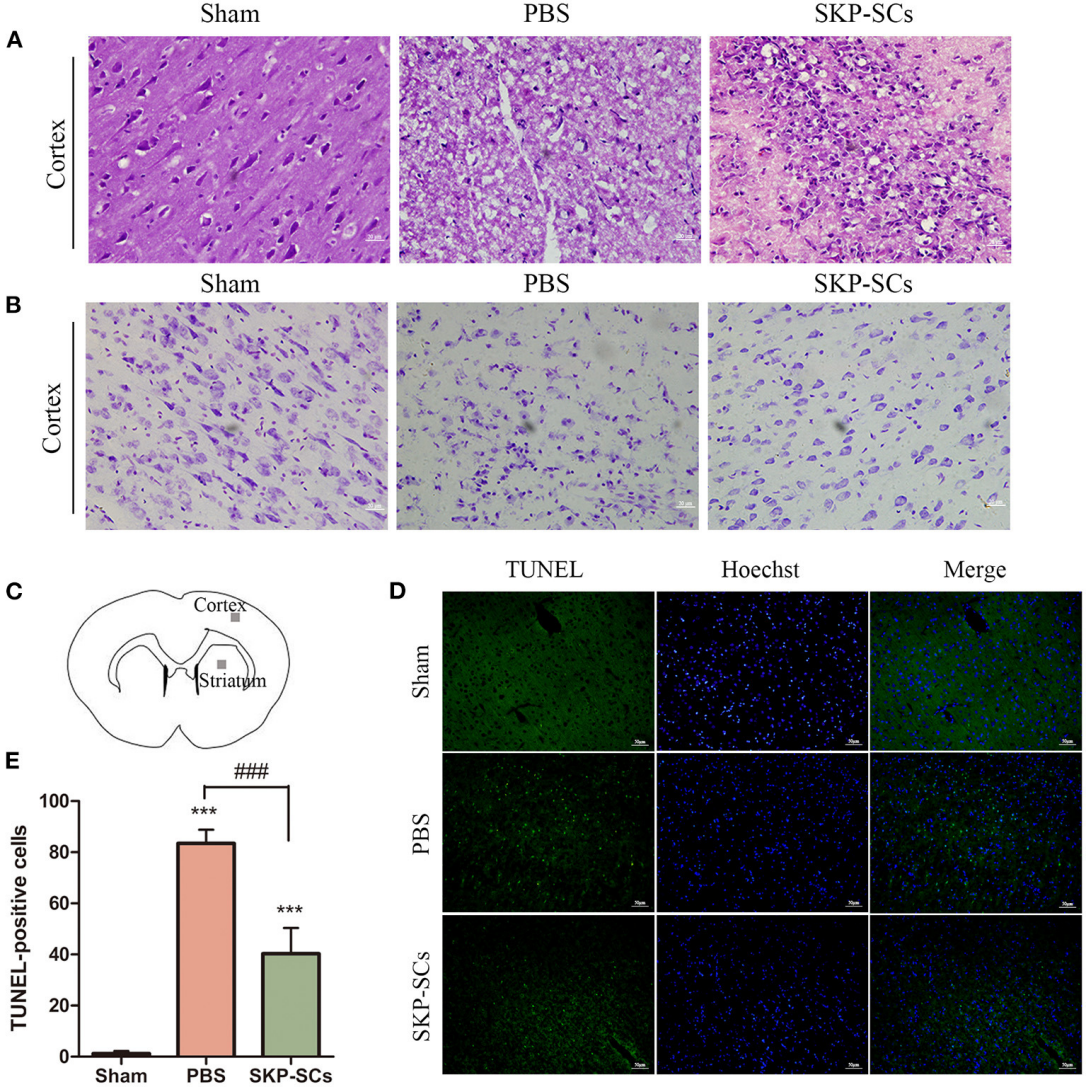
Protection of cortical neurons by SKP-SCs in the rat MCAO model. **(A)** H&E staining of the rat brains in the Sham, MCAO+PBS, and MCAO+SKP-SCs groups on Day 4 after transplantation. SKP-SCs remarkably improved survival and organization of the cortical neuron. **(B)** Nissl staining confirmed H&E staining results. **(C)** Cerebral cortex and striatum were sampled for the quantitation of TUNEL-positive cells. SKP-SCs significantly reduced TUNEL-positive cells in both the cortex and the dorsal striatum on Day 4 **(D,E)**. *N* = 3, ***P < 0.001 compared with Sham group. ^*###*^*P* < 0.001 compared to MCAO+PBS group. Scale bars = 50 μm.

The neuronal survival and morphological changes in the penumbra region were analyzed with H&E staining and Nissl staining on Day 4. Comparing with rats with sham surgery (Figure 5A, Sham), MCAO rats with PBS control suffered a significant loss of neurons in the cerebral cortex. Cytoplasm in the disorderly arranged neurons was condense and the nuclei of many neurons were darkly stained, shrunk or dissolved. Moreover, evident interstitial edema was observed (Figure 5A, PBS). SKP-SCs appeared to remarkably improve the cortical histology. There was an increased number of morphologically healthy neurons. Cytoarchitecturally, neurons appeared to be more organized (Figure 5A, SKP-SCs). Histopathological results with Nissl staining were consistent with H&E findings (Figure 5B, Sham, PBS, SKP-SCs).

Neuronal apoptosis was assessed by detecting TUNEL-positive cells in the penumbra region of the cortex and the striatum (Figure 5C). On Day 4 following MCAO, compared with the PBS control group, SKP-SCs transplantation remarkably reduced TUNEL-positive cells (*p* < 0.001) (Figures 5D,E).

### SKP-SCs CM Promoted Neuronal Survival in the *in vitro* OGD/R Model

**Figure 6:**
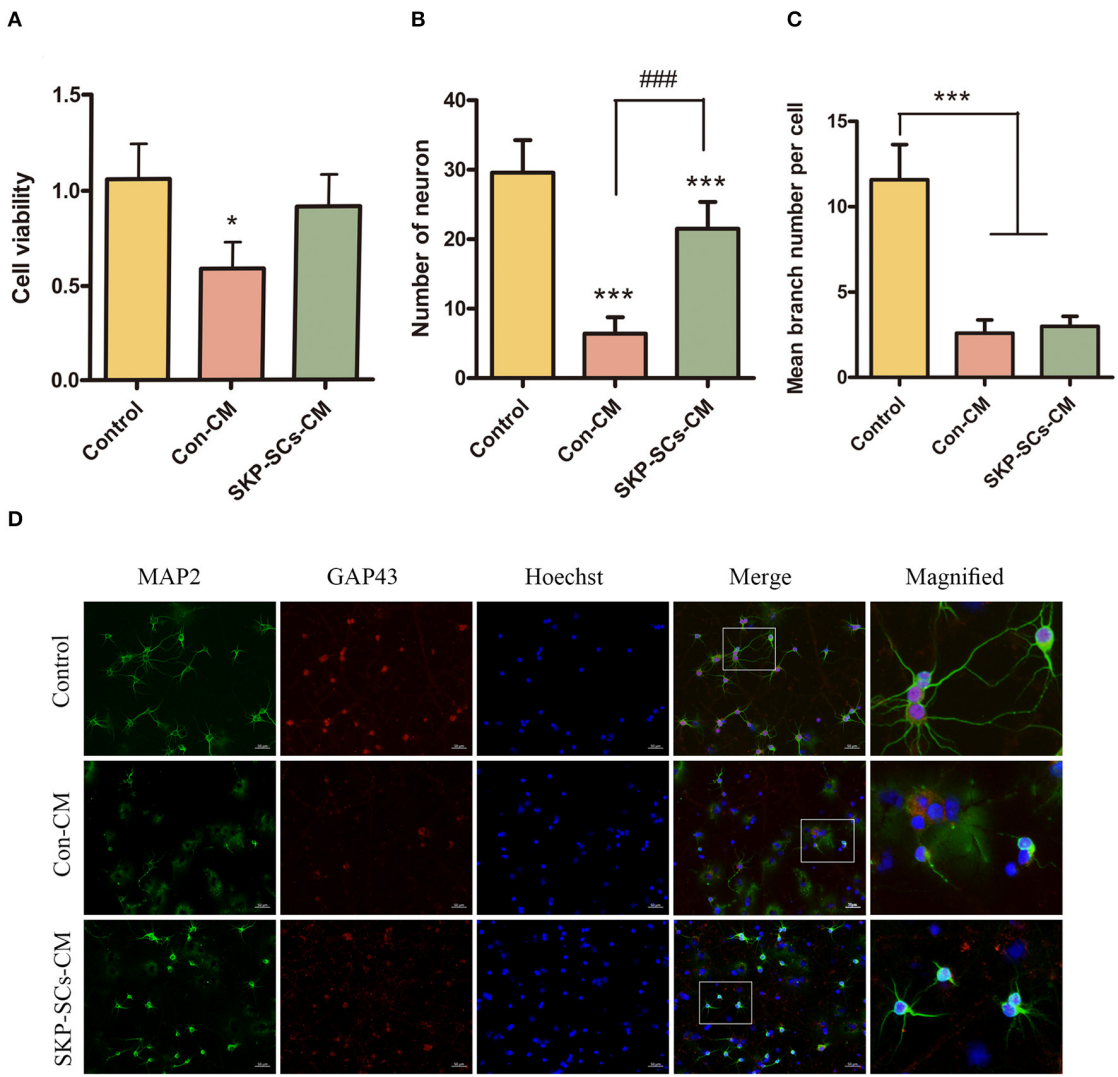
SKP-SCs promoted neuronal survival in the *in vitro* OGD/R model using cultured primary cortical neurons **(A,B)**. SKP-SCs CM improved cell viability **(A)** and total number of live cells **(B)** but did not appear to increase numbers of neuronal branches **(C,D)**. The white squares on Merge images **(D)** represent the areas magnified and presented as the image on the right. Scale bars = 50 μm. *N* = 3. *p < 0.05, ***p < 0.001 compared with control medium group. ^*###*^*p* < 0.001 compared to control medium group.

SKP-SCs CM appeared to improve the survival of PCNs after oxygen/glucose deficiency assault. Cell viability revealed by CCK-8 was significantly better than PCNs cultured with control medium (*p* < 0.05). The viability of SKP-SCs CM cultured PCNs appeared to be comparable to that of PCNs without any oxygen/glucose deficiency (Figure 6A). SKP-SCs CM not only improved the viability of the PCNs, but also increased the number of surviving neurons (*p* < 0.001) (Figure 6B). However, SKP-SCs CM did not appear to improve neurite branching as demonstrated by MAP2 and GAP43 staining (Figures 6C,D).

### SKP-SCs CM Inhibited OGD/R-Induced Neuronal Apoptosis

**Figure 7:**
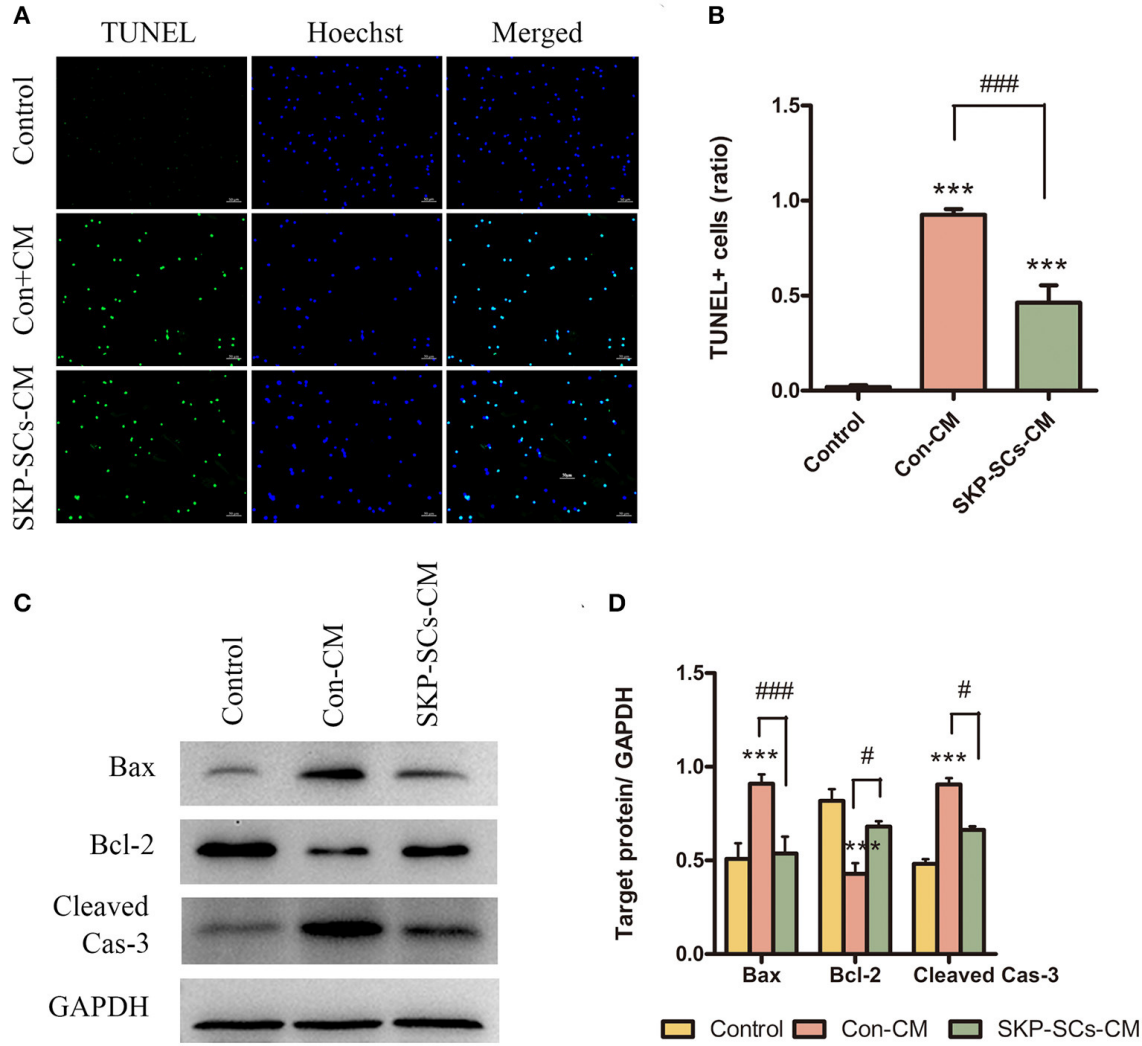
SKP-SCs inhibited OGD/R-induced neuronal apoptosis *in vitro* **(A,B)**. SKP-SCs decreased expression of Bax and cleaved caspase-3 but increased the expression of Bcl-2 **(C,D)**. Scale bars = 50 μm. *N* = 3. *N* = 3. ***p < 0.001 compared to Control group. ^#^*p* < 0.05, ^*###*^*p* < 0.001 compared with control medium group.

The number of TUNEL-positive cells was markedly reduced by SKP-SCs CM compared with the control (Figures 7A,B). Protein levels of Bax, and cleaved caspase-3 were statistically elevated after exposure to OGD/R (*p* < 0.001). This increase, however. was remarkably inhibited by the addition of SKP-SCs CM (*p* < 0.001 for Bax, *p* < 0.05 for Cleaved Cas-3) (Figures 7C,D). On the contrary, OGD/R decreased the level of Bcl-2, and this decrease was reversed by SKP-SCs CM (*p* < 0.05) (Figures 7C,D).

## Discussion

SKP-SCs have been previously shown to promote repair and functional recovery in rats with spinal cord injury (9, 12, 13, 18). SKP-SCs could also protect SH-SY5Y cells against 6-OHDA-induced neurotoxicity (15). In the current study, a rat *in vivo* MCAO model and an *in vitro* primary cortical neuron culture with OGD/R model were used to explore whether SKP-SCs cell therapy promotes neuroprotection after AIS. It was found that SKP-SCs can survive and maintain their phenotype up to 4 weeks. Intracarotid transplantation of SKP-SCs effectively migrated to the periinfarct area, inhibited neuronal apoptosis, reduced infarct volume, and improved functional recovery in the MCAO rats. Moreover, SKP-SCs CM inhibited OGD/R-induced neuronal apoptosis and promoted neuronal survival in the peri dish. Given the favored extended time window, our study has provided the first evidence that SKP-SCs could be a potential novel cell therapy for patients with AIS, in addition to the current standard of care.

The delivery route and the optimal time point for SKP-SCs treatment are two of the important issues that must be addressed in future clinical development. As described in previous studies (17), many delivery routes have been tested in experimental stroke models, including intra-arterial, intravenous, intraventricular, intracerebral, and intra-nasal, with the first three being the most commonly used. Compared with local intraventricular transplantation, systemic transplantations (intra-arterial or intravenous) to achieve high-dose stem cell injection in a low-invasive manner are relatively safer and highly efficacious. Studies showed that systemically transplanted stem cells performed better in anti-inflammatory, brain remodeling, and induction of neuroprotection for neurological diseases in the acute and plateau phases than local delivery (19, 20). Underperformance of targeted cell dosage by intravenous route was usually due to the inevitable long-distance migration and certain organs' filtering effect. Multiple studies suggested that up to 80% of injected cells by intravenous approach were distributed in various non-targeted tissues, most of which were trapped in the lungs, followed by liver and spleen (21–23). For ischemic stroke, the neuroprotective effect of stem cell transplantation strictly depends on the number of cells implanted into the ischemic lesion (19, 24). Intra-arterial injection is regarded to be safer and more feasible to target damaged brain. It is important to note that as mechanical thrombectomy has become the standard of care for the treatment of acute ischemic stroke, intra-arterial approach is gaining ground in clinical translation (25). To align with this, we chose to infuse SKP-SCs through carotid artery catheter when cerebral perfusion was restored in MCAO rats. In regard to optimal time when stem cells should be transplanted, early application of cell therapy was found to effectively protect and repair neurons (26), and many studies have reported improvements of outcome with cell therapy given within 24 h after AIS (1, 2, 27–29). However, stem cell transplantation immediately after reperfusion has not been documented. Our data have provided the first evidence that intracarotid transplantation of SKP-SCs immediately after reperfusion could also effectively inhibit neuronal apoptosis and improve functional recovery, suggesting that stem cells could be introduced via the arterial route to the infarcted region immediately after thrombectomy.

The ability for SKP-SCs to survive and migrate *in vivo* is of vital importance (30), especially in the area near the infarct core. The brain area adjacent to the infarct core is a key area for neural rehabilitation due to its strong neuroplasticity (31). Results in the current study showed that SKP-SCs effectively migrated to the area around the infarct core after intracarotid infusion but were rarely detected in the infarcted core itself and other intact brain regions. The exact mechanism of this migration/survival pattern remains to be explored. Possible reasons may include the barren environment of the infarcted core not suitable for the survival of SKP-SCs, and the intact blood-brain barrier of normal brain regions difficult for SKP-SCs to penetrate. SKP-SCs appeared to be able to survive in the periinfarct area for a long time, at least for 4 weeks as revealed in the current study. This observation is consistent with previous reports (10). SKP-SCs in this study were isolated from the dorsal skin of the rat. Studies have shown that murine SKPs from dorsal skin originate from mesoderm mesenchyme non-neural crest-derived cells (14). These mesenchymal-derived SKPs can, without genetic manipulation, differentiate to functional neurons (7, 8) Schwann cells (13, 14), or osteocytes (9), suggesting that developmentally defined pedigree boundaries are more flexible than commonly thought (14). Studies have shown that the regenerating peripheral nerves and neonatal trembling central nervous system were able to guide naive SKPs to produce myelin Schwann cells (9). Importantly, environmental cues may induce selective differentiation of SKPs. In the current study, SKPs responded to neural crest cues including forskolin, heregulin-1β and N2 supplement to generate functional Schwann cells.

Although multiple studies have reported significant efficacies of stem cells in stroke, the mechanism of action of stem cell transplantation remains explored. Although transplanted stem cells were unlikely to directly help reshape neural circuits by integrating dead or dying neurons (32, 33), studies have shown that amplification of endogenous repair mechanisms in the brain, such as the proliferation and migration of endogenous neural stem cells, may contribute to the positive effects of stem cells therapy (1, 6, 30, 34). It is proposed that after migration to the ischemic environment, the transplanted stem cells resemble micropumps and continuously secrete trophic factors and other mediators, creating a microenvironment that supports endogenous neuroblasts to survive and help with tissue remodeling. For example, in the CNS, insulin-like growth factor-2 (IGF-2) is a critical factor for neuronal growth and survival. IGF-2 was found to be able to preprogram maturing macrophages to acquire oxidative phosphorylation-dependent anti-inflammatory properties (35). Our previous study demonstrated that IGF-2 expression was much higher in SKP-SCs CM than in the control medium (15). On the other hand, tissue microenvironment could also have a significant impact on the properties of the stem cells. A recent study found that proinflammatory factors altered the secretome and immunomodulatory properties of human SKPs (36). Altered SKP-SCs could in turn secrete more factors to impact on nearby neurons or glia cells. Further studies are warranted to explore all possible mechanisms.

## Conclusion

The current study revealed that SKP-SCs mediated a neuroprotective effect via the inhibition of apoptosis in *in vivo* and *in vitro* models of AIS. SKP-SCs are autologous, easy-to-obtain, and expandable source that can be reliably induced into Schwann cell-like precursors. In combination with endovascular thrombectomy, intracarotid artery transplantation of SKP-SCs during reperfusion could serve as a feasible auxiliary therapy for the treatment of acute ischemic stroke.

## Data Availability Statement

All data generated and/or analyzed during this study are included in this published article.

## Ethics Statement

The animal study was reviewed and approved by the Experimental Animal Center of Nantong University.

## Author Contributions

JL performed the experiments (behavioral tests, etc.), analyzed data, prepared the figures, and wrote the manuscript. RC performed the experiments (behavioral tests, etc.) and contributed to the analysis and interpretation of data. JW performed the experiments (animal model, etc.). KK designed the study and critically reviewed the manuscript. JS, YC, and MC supervised the project and revised the manuscript critically. All authors read and approved the final version of the manuscript.

## Conflict of Interest

The authors declare that the research was conducted in the absence of any commercial or financial relationships that could be construed as a potential conflict of interest.

## Abbreviations

CM: conditioned medium
DMEM: Dulbecco's modified eagle medium
GAPDH: glyceraldehyde-3-phosphate dehydrogenase
GFAP: glial fibrillary acidic protein
MCAO: middle cerebral artery occlusion
mNSS: modified Neurological Severity Scores
OCT: optimal cutting temperature compound
OGD/R: oxygen and glucose deprivation/reoxygenation
PBS: phosphate buffer saline
PCNs: Primary Cortical Neurons
PVDF: polyvinylidene fluoride
SKP: skin-derived precursors
SKP-SCs: SKP-derived Schwann cells.
