# Supplementary figures and images for: Intracarotid Transplantation of Skin-Derived Precursor Schwann Cells Promotes Functional Recovery After Acute Ischemic Stroke in Rats
Source: Front Neurol. 2021 Feb 4;12:613547. doi: 10.3389/fneur.2021.613547 (PMC7902026; doi:10.3389/fneur.2021.613547)

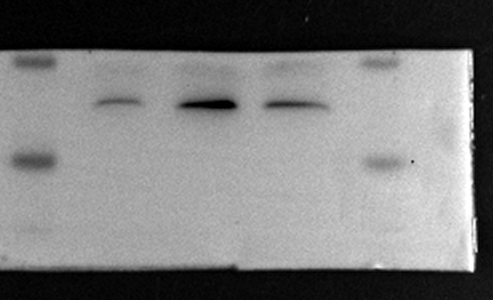

Supplement: Supplementary file 1 [file Image_1.TIF]

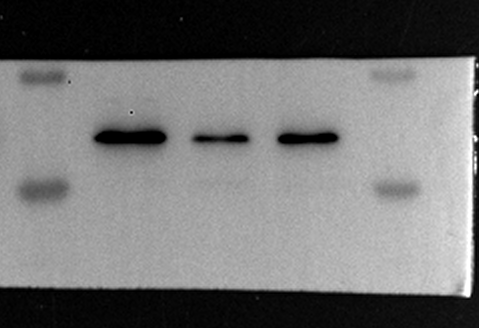

Supplement: Supplementary file 2 [file Image_2.TIF]

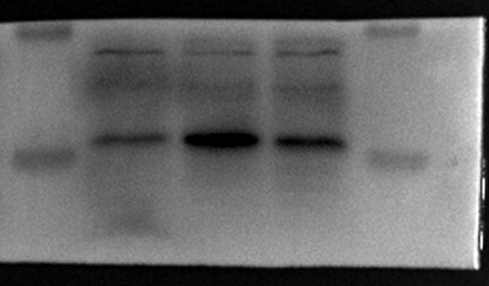

Supplement: Supplementary file 3 [file Image_3.TIF]

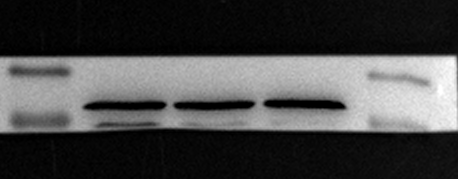

Supplement: Supplementary file 4 [file Image_4.TIF]
